# Multiple Identifications of Employees in an Organization: Salience and Relationships of Foci and Dimensions

**DOI:** 10.3390/bs12060182

**Published:** 2022-06-07

**Authors:** Andrey V. Sidorenkov, Eugene F. Borokhovski, Wladimir A. Stroh, Elena A. Naumtseva

**Affiliations:** 1 Academy of Psychology and Education, Southern Federal University, 105/42 Bolshaya Sadovaya Str., Rostov-on-Don 344006, Russia; avsidorenkov@sfedu.ru; 2 Centre for the Study of Learning and Performance (CSLP), Concordia University, 1515 St. Catherine Street West, S-GA-2.126, Montreal, QC H3G 1W1, Canada; 3 Faculty of Social Sciences, National Research University Higher School of Economics, 20 Myasnitskaya Str., Moscow 101000, Russia; vstroh@hse.ru; 4 LLC SN Consulting, 4 Kamchatskaya Str., Moscow 107065, Russia; yelena_naumtseva@mail.ru

**Keywords:** personal identification, interpersonal identification, micro-group identification, group identification, sub-organizational identification, organizational identification

## Abstract

This research addresses: (1) the salience of employees’ social (organizational, sub-organizational, group, micro-group), interpersonal, and personal identifications and their dimensions (cognitive and affective); (2) and the relationship and structure of the identifications of employees in different areas of professional activity. The study was conducted on independent samples of employees in the socio-economic sphere (241 participants), in the law enforcement agency (265), and in higher education (172). To assess the respective identification foci and dimensions, the study employed four questionnaires. The personal identification was the weakest and the micro-group identification was the strongest for both dimensions in all samples. The affective dimension prevails over the cognitive in all identifications, except for interpersonal. Social identifications were significantly positively correlated to each other in all samples whereas personal identification was significantly negatively correlated with all social identifications (on the affective dimension) in two samples. The results expand our understanding of the identifications of employees in organizations.

## 1. Introduction

Over the past three decades, research has attended to the identification (ID) of employees with collective actors (primarily with an organization and a small group) as well as with individual actors (colleagues or a supervisor) in an organizational context. This interest is largely based on practical considerations, since IDs can affect other attitudes and behaviors of employees, the effectiveness of individual and group work, etc. Van Knippenberg and Van Schie [1] note that our understanding of organizational attitudes and behaviors can greatly benefit from a serious attention to the plurality of ID foci. For instance, it was discovered that the organizational ID of employees positively correlates with organizational commitment, job involvement, occupational attachment and work group attachment, in-role and extra-role behaviour, and is negatively correlated with intention to leave [2,3,4] and counterproductive work behaviors [5], and has non-linear relationships with health and burnout of employees [6].

In turn, group ID and interpersonal ID (with a supervisor and colleagues) are positively related to organizational civic behavior, job satisfaction, group morale [7,8,9,10,11], knowledge sharing [12], known to decrease relationship conflicts within work groups [13], etc. Not only individual IDs, but also the interactions among them and their ratio are capable of creating such effects. For example, it has been found that combined high levels of group ID and organizational ID are more closely associated with organizational citizenship behavior (OCB) than either of them alone [14], or when both IDs (or at least one of them) are low [15]. There has also been observed some disparity between organizational and group IDs, as the former strengthens intergroup consensus, whereas the latter weakens it [16].

Some identifications—i.e., sub-organizational (ID with a secondary-level structural unit) [17], micro-group (ID with an informal subgroup within a small group) [18] and personal [19]—have received lesser attention than others. At the same time, it has been shown that the stronger the group ID, on the one hand, and the weaker the micro-group ID and personal ID, on the other hand, the stronger manifestations of the offering of quality ideas and suggestion by employees within the group [20].

In this article, we intend to consistently use the terms: (a) “foci”, when referring to identifications with certain actors (e.g., organization as a whole or a work group); and (b) “dimensions”, when referring to different facets of a particular identification (e.g., cognitive and affective) [1,21,22]. However, we acknowledge the existence and occasional use of other equivalent/synonymous terminology, namely: “levels” [23] and “components” [23,24,25], respectively.

A comprehensive study of the entire spectrum of foci and dimensions of employees’ ID is also of a fundamental scientific value, as it contributes to the expansion of our understanding of self-concept components: collective self and personal self [26]. The former touches on social IDs, and the latter on personal ID. For many years, psychologists have viewed ID in dualistic terms: as social vs. personal, collective vs. individual [27], etc. However, social ID in an organizational context can have several foci (depending on which particular collective actor it focuses on): organizational, sub-organizational, group, and micro-group IDs. They contribute to the fulfillment of a wide range of needs, such as belonging [28], self-enhancement [29], and self-verification [30], etc. Thus, different identifications imply different levels of self and perspectives of individual’s self-determination.

There are some gaps in the study of the multitude of foci and dimensions of employee’s IDs within the organization. First, researchers tend to analyze relative strength of ID with the examples of organizational and group IDs [21,31]. Some authors theorize about the relative salience of social and personal IDs, in the broader sense of conditions they may depend on [32]. However, there is virtually no empirical evidence of how salient different ID foci are in comparison with each other: social (organizational, sub-organizational, group, and micro-group identification), interpersonal, and personal.

Second, though some experts do pay attention to different ID dimensions (e.g., cognitive and affective), they also limit this interest to organizational and group IDs [25,33]. However, other previously listed IDs, we assume, may have similar dimensions. Moreover, it is difficult to find empirical studies that simultaneously examine the relative strength of the different dimensions of all ID foci.

Third, also of interest are relationships (type and strength): (a) among social IDs—organizational, sub-organizational, group, and micro-group; b) between each social ID, on the one hand, and interpersonal and personal IDs, on the other. Researchers either theorize about the relationship between social and personal IDs [32], or in empirical research, occasionally pay attention to the relationship between organizational and group IDs [15,34] and, in very rare cases, to the relationship between organizational (group) and interpersonal IDs [35,36], among group, micro-group and interpersonal IDs [37]. However, a complete and empirically grounded picture of the relationships among all ID foci for each of their dimensions is virtually non-existent.

The current research is complex in its nature and aims at closing the above-mentioned gaps by means of addressing the following. (1) A wide spectrum of employees’ IDs in an organization, namely: (a) organizational ID; (b) sub-organizational ID; (c) group ID; (d) micro-group ID; (e) interpersonal ID; and finally, (f) personal ID. (2) Two ID dimensions—cognitive and affective—of each ID focus.

We formulated the following research questions that guided our study.

1. What is the comparative (i.e., relative to each other) salience of different ID foci (for each dimension)?
2. Is there a difference in strength between ID dimensions—cognitive and affective—of each of the ID foci? If so, which of the ID dimensions are significantly stronger?
3. What is the nature and degree of association among ID foci (for each dimension)?
4. What structural model could plausibly explain the relationships among ID foci and dimensions?

Addressing these questions in the current research on a variety of samples would allow us to identify and study both the generic and specific characteristics of strength of relationships among IDs.

The literature review that follows features three sections. The first describes a conceptual multidimensional model of employees’ identification in an organizational context, which includes several foci and dimensions. This model lays out a conceptual foundation for our study. In the second section, we substantiate the salience of and interrelations among different foci and dimensions of identification and formulate the corresponding hypotheses that guided the study. In the third, we analyze and draw on the available research literature that addresses the relationships among different social IDs, between social IDs and interpersonal ID, as well as between social IDs and personal ID, and put forward some assumptions about patterns in them. In addition, this section pays special attention to the structure of all foci and dimensions of identification.

## 2. Multidimensional Conceptual Model of Employees’ IDs

**Figure 1:**
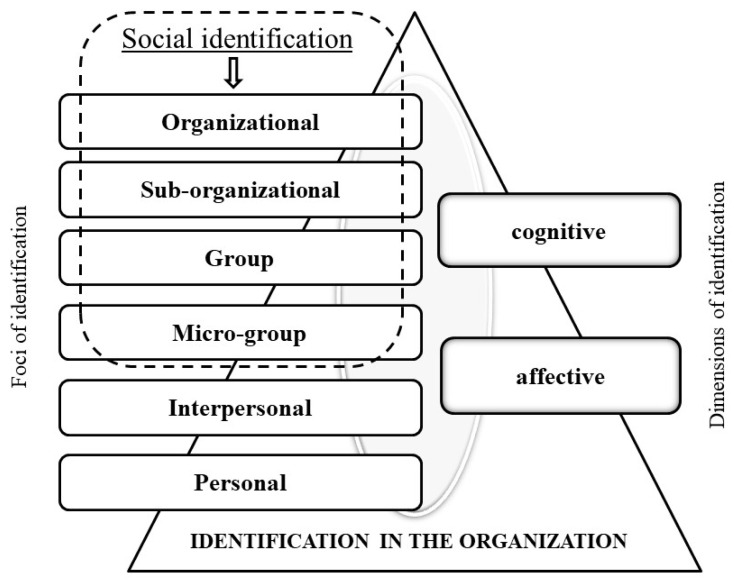
A Multidimensional Conceptual Model of Employee ID in an Organization.

Thus, we offer a multidimensional conceptual model of employees’ IDs in an organization (Figure 1), which is based on two dimensions: (1) foci of ID (depending on the foci of ID, which can be a specific individual or collective)—organizational, sub-organizational, group, micro-group, interpersonal, and personal; and (2) dimensions of ID (depending on aspects of the relationship to the object of ID)—cognitive and affective. A cognitive dimension is an individual’s sense of ownership of a significant subject (individual or collective) and integrative connection with the individual, a perception of oneself or another in accordance with one’s actual characteristics, while an affective dimension is an individual’s experience of connection with oneself or with another and assessment of the positivity of this association, the reliving of one’s correspondence to oneself or another and the events occurring with it [18]. Thus, each ID focus includes two dimensions.

This model has some limitations. In full, it can be extended to organizations wherein within the structure there are all levels, i.e., small groups (teams, etc.) and structural units (departments, etc.) present. If any organizational level is missing, then the corresponding ID focus is excluded from the model. In addition, there may be another ID focus that is not represented in this model. For example, transnational corporations contain subsidiaries/branches. Identification with those branches could be considered analogous to identification with the corporation as a whole. Additionally, if a small group does not contain a single informal subgroup, which, in fact, is extremely rare (Sidorenkov et al., 2014), then micro-group ID will be absent. In turn, both ID dimensions—a cognitive and an affective one—are the integral attributes of any foci of identification. It is difficult to imagine an individual perceiving a relationship without experiencing it, and vice versa.

## 3. Relative Strength of Foci and Dimensions of Employee Identification

### 3.1. ID Foci

Researchers often pay attention to organizational ID: its antecedents [38,39,40,41], consequences [42,43,44,45], and the moderating and mediating role it plays [46,47,48,49]. However, focusing only on organizational ID can lead to serious omissions in studying the role of ID in the processes and results of individual and collaborative activities [1]. It is necessary to consider the many IDs associated with membership in the organization.

Therefore, specialists also study, but less frequently, the identification of employees with their working group or team [1,33,50]. Studies have shown that identification with a working group is stronger than identification with an organization [1,21]. Summarizing the points of view of various authors, Riketta and Van Dick [21] identified several explanations for this difference between organizational ID and group ID: (a) professional activities of people occur mainly in working groups, and thus the relationships among members of the in-group will be closer than between employees from different groups in the organization; (b) in-groups have a stronger psychological influence on their members than the organization; (c) the in-group is more distinct for its members than the outgroups in the organization and the organization as a whole; (d) many groups (teams) in the organization have relatively high autonomy and authority, and thus the working group is more significant for its members than the organization as a whole. Additionally, according to Brewer [51], individuals strive for an optimal balance between being considered a unique person and being recognized as a group member. That is, people want to stand out and fit in at the same time. This balance is best achieved by belonging to smaller groups rather than larger groups. As such, this tendency should reinforce the group ID, not the organizational ID [1]. However, we should not assume that group ID is always stronger than organizational ID. There is a possibility that individual differences [52] or diversity among organizations [53], would determine the reversed predominance of one over the other.

In organizations with a multi-level structure, in addition to identifying employees with an organization and a small group, there may be identification with a secondary-level structural unit (for example, a manufacturing facility at a factory or a department/faculty at a university), in which a group is included. This identification can be termed sub-organizational ID (or work-unit identification [54]). The magnitude of this ID may depend on many factors: group ID, the number of groups and the number of employees in the unit, the competition of the group with other groups in the unit, the specific culture in the unit, which positively or negatively differs from the culture of the organization as a whole, etc. Therefore, we can assume that sub-organizational ID in some cases can be equal in strength to organizational ID and group ID [54], and in others it can be stronger than organizational ID and weaker than group ID. For example, strong group ID and competition between groups in a unit is more likely to lead to a decrease in sub-organizational ID.

Specialists rarely focus on informal subgroups in small groups. Sometimes the concept of “subgroup identification” is used in the literature, but in fact they mean identification either with a working group in an organization [55] or with a subcontractor [17]. We understand an informal subgroup as a set of group members that are united on the basis of one or more general psychological and significant signs among themselves, compared to those among other members of the group. It was found that in small working groups of five or more people, there were from one to four subgroups; on average, more than half the members are included in subgroups [18]. Most common were dyads in groups, triads were often found, and subgroups of four and five people were the least likely to arise. Therefore, members can be identified not only with the in-group, but also with informal subgroups within the group (micro-group ID). It has been shown that micro-group ID among employees is significantly stronger than group and interpersonal IDs [18]. To explain the predominance of micro-group ID over group ID, some of the arguments identified by Riketta and Van Dick [21] in their analysis of higher group ID compared to organizational ID, could be employed.

Organizational, sub-organizational, group, and micro-group IDs can be combined with such a general concept as “social identity” (collective self) [26]. This is consistent with the notion that people have as many social identities as there are groups to which they feel they belong [56]. Social IDs vary in their subjective significance for individuals, in their stable and situation-dependent manifestation. The picture of relative strength and prevalence of different social IDs may change depending on some factors, such as the specifics of working conditions and the content of professional activity. The perception by employees of the similarities of the collective entities with which they are identified, and the particulars of the interaction situation of their team or group (i.e., competition, cooperation or lack of active interaction) with other teams are also important factors. The positive relationship between organizational and group IDs increases when employees perceive the high similarities between their organization and their work group [34]. The disintegrative interaction (for example, competition) of a small group with other groups in the organization will strengthen group ID and weaken micro-group, sub-organizational and organizational IDs. In the absence of intensive intergroup interaction, micro-group ID will be stronger than group ID (in this case, the salience of sub-organizational ID and organizational ID is not obvious, since it depends on a number of additional conditions).

People can identify not only with some collective actors (organization, unit, etc.), but also with some individual members: colleagues [9] and a leader/supervisor [10,57], which implies interpersonal ID. In other words, the employee can feel a connection with another individual who has characteristics that are significant for him (for example, the manner of dressing, the characteristics of interpersonal interaction or behaviour in difficult situations), which he does not possess or insufficiently expresses. It was found that in small groups, interpersonal ID is expressed in much the same way as group ID, but significantly weaker than micro-group ID [18].

Employees have a personal identity (personal self), which consists of self-determination in terms of the physical, intellectual, moral and other qualities of a person, characterizing him as a unique person in terms of individual differences from other people in the group [26,58]. If personal ID is the most salient, then a person has, firstly, a personified perception of himself and others (perception through the prism of individual characteristics), and secondly, egocentrism (a person thinks, experiences and acts in accordance with his individual goals, needs and traits). Personal ID, like social ID, in its core, is based on the relationship of the individual, but primarily with him/herself, and not with some collective subject. Unfortunately, researchers rarely pay attention to personal ID in an organizational context [22].

Another question is the salience of personal ID compared to other employee IDs in the organization. It can be assumed that, in the majority of employees, personal ID is weaker than social IDs—organizational, sub-organizational, group, and micro-group. A person is included in an organization, division, group, and possibly in an informal subgroup, and therefore will experience their significant influence. A person must adjust to meet the expectations, requirements and norms of a certain collective actor, which he is included in, as the opposite of being guided exclusively by own ideas, interests and needs. However, the balance (prevalence) of personal ID and social ID may be explained by various factors, including the specific characteristics of the organization. For instance, in law enforcement, where work and communication are strictly regulated, personal ID tends to be suppressed and therefore weaker, whereas social IDs and interpersonal ID would be stronger than those of employees of business companies and higher educational institutions.

**Hypothesis** **1** **(H1).** *Of all IDs, micro-group ID is the strongest and the weakest is personal ID.*

### 3.2. ID Dimensions

Some researchers understand social ID (primarily by the example of group and organizational IDs) as a multidimensional construct that includes several dimensions, for example, cognitive, affective and evaluative [59], cognitive, affective, and behavioural [18,24], or cognitive, affective, evaluative, and behavioural [33,60]. The discussion on structural composition of the ID dimensions continues with the prevalent arguments in favor of a two-fold structure [22]. The cognitive ID dimension is present in many definitions and descriptions of identification [53,61]. However, other authors do not doubt the existence and importance of the affective dimension. As Harquail [62] figuratively put it, identification “…engages more than our self-categorization and our cognitive brains, it engages our hearts…” (p. 225). In order for an individual to form an internal psychological connection with an organization (group, etc.), he must not only feel, but also experience this connection.

Other dimensions are controversial. For example, it can be argued that the evaluative dimension is part of the affective dimension, since the experiencing of and response to any event is always based on its appraisal by the individual. Some researchers believe that behavior should be viewed as a likely outcome of ID rather than as its essential dimension [27]. This point of view is quite prominent in the theory of self-categorization [58], according to which a high level of social ID leads to depersonalization, which, in turn, produces group behavior, i.e., behaviour largely aligned with the group’s key attributes. We think that, since ID is a certain relationship of an individual to another individual or collective subject, behavior is more likely to be a consequence of the relationship, and not just a part of it.

In our opinion, not only social ID (for example, organizational and group), but also interpersonal ID and personal ID include the corresponding dimensions. Knowledge of these dimensions better orients us in the search for answers to questions of either conceptual or applied nature. First, since both dimensions constitute the essential fabric of identification, what is the relative (that is, in comparison to each other) strength of each dimension in each focus of identification? The answer to this question will make it possible to understand how stable and deep the connection to (identification with) a collective or an individual actor is, and to what extent the individual will assimilate the key attributes of the actor with whom he identifies himself. In our opinion, the clear predominance (in strength) of the affective dimension will indicate a higher stability and depth of the ID.

Second, knowing the strength of each dimension of a particular focus of ID, can we predict its consequences? For example, the affective dimension of some ID foci has been shown to be a stronger (than their cognitive dimension) predictor of individual’s contribution to collaborative activities in groups [23], interpersonal helping and promotion of loyal boosterism [44].

**Hypothesis** **2** **(H2).** *Employees have a more pronounced affective than cognitive dimension at all foci of ID.*

### 3.3. The Relationship among ID Foci

#### Connections among Social IDs, and between Social IDs and Interpersonal ID

A significant positive linear relationship has been found between organizational ID and group ID [15,34,50], between organizational ID and sub-organizational ID [54], between micro-group ID and ID with peers [37], and between organizational ID and ID with the supervisor [35,36,63]. In particular, it was shown that the identification of employees who tend to follow the lead of the leader plays a decisive role in the subsequent formation of collective identifications—organizational and group IDs [35].

We believe that under normal circumstances (i.e., without any explicit disintegrative interaction among structural units, for example, between work groups or between secondary-level structural divisions), there will be a positive relationship among micro-group, group, sub-organizational, and organizational identifications. This is due to the fact that all of them, although having different foci, relate to the same class of social identifications. Some of them are embedded or nested within others in the chain of structural levels of the organization: subgroup-group—secondary-level structural division—organization as a whole. In addition, there is likely to be a positive relationship between the interpersonal ID and all social IDs. This can be explained by the fact that the identification of workers with structural units or the entire organization can be the basis for their interpersonal ID. In turn, the identification of some employees with other workers (also included in a subgroup, group, etc.) can be projected onto their identification with the corresponding structural units or the entire organization. However, the pair-wise links between some social IDs, between interpersonal ID and social IDs, may vary (be absent or be negative) depending on specific working conditions, the content of professional activity, etc.

Therefore, we can assume that in the usual conditions for the functioning of the organization and the work group (without obvious competition between groups and units):

**Hypothesis** **3a** **(H3a).** *There are positive relationships between social IDs—organizational, sub-organizational, group, and micro-group.*

**Hypothesis** **3b** **(H3b).** *There are positive relationships between interpersonal ID and all social IDs.*

### 3.4. Connection between Personal ID and Other IDs

There are differing points of view on the relationship between social ID and personal ID. According to the theory of self-categorization, there is a mutual exclusion between the strengths of social ID and personal ID. The categorization of oneself as an individual “blocks” the perception of oneself as a member of a group, while the categorization of oneself as a member of an in-group different from a member of an out-group reduces the perception of oneself as a unique individual [26]. In support of this assumption, there is evidence that, for example, self-stereotyping is the result of a pronounced social identity [64,65,66]. Another point of view is based on the idea of complementarity of social and personal identities in the structure of Self [67], and their simultaneous manifestation [68]. The latter point is largely supported by Ashforth and Schinoff [32], who also argue that there is a dynamic tension between personal and social identities (people want to be unique and be part of a collective).

We guess that both points of view can take place depending on a number of circumstances, for example, on the specifics of the profession and the ID dimension. In creative professions (for example, programming, design and scientific work), involving individual manifestations, between these identities there will probably be a relative independence in the cognitive dimension and a negative relationship in the affective dimension. In organizations with a rigid vertical of power, the principle of unity of command, strict observance of subordination, etc. (for example, in the army and police), there is more likely to be a linear negative relationship between personal ID and social IDs (organizational, sub-organizational and group) for both dimensions. In small groups with a high interdependence of tasks and collective responsibility for the overall result of activities, group ID can suppress personal ID. In turn, in-groups with low interdependence of tasks, and a prevalence of individual results and individual responsibility, there may not be a connection between group ID and personal ID.

**Hypothesis** **4** **(H4).** *The relationship between personal ID and interpersonal, micro-group, group, sub-organizational and organizational IDs depends on their dimensions and the specifics of the professional activities of employees.*

### 3.5. Structure of ID Foci and Dimensions

It is necessary to study not only the relationships between specific ID foci separately, but also the structure of all foci and dimensions in an association. Earlier, we suggested that personal ID, for one, is weaker than other identifications, and also, either unrelated or negatively related to other identifications—depending on the specific ID dimension and on the employees’ professional sphere. Therefore, personal ID can occupy a separate place in the structure of employees’ IDs. In turn, social IDs may coincide or differ in their importance for employees. Among them, micro-group ID occupies a special place. This is due to the fact that in most cases there is, firstly, no clear competition for the in-group/unit with other groups/units; and secondly, when the group has informal subgroups, it is more significant for people, as opposed to other social IDs. In addition, many employees perceive a noticeable difference between the group and the secondary-level unit and organization, and at the same time, the similarities between the latter two. This tendency is not absolute and may vary depending on the characteristics of relationships and working conditions within the organization—something that has previously attracted our attention while we were addressing the issue of interconnections among different social identifications.

**Hypothesis** **5** **(H5).** *In the structure of employees’ identifications, personal ID is a relatively autonomous unit across the areas of professional activity. In turn, interpersonal, micro-group, group, sub-organizational and organizational IDs may form different configurations (integrating or separating from one another) depending on the specific professional field.*

## 4. Method

### 4.1. Sample

Our study is conducted on three independent samples. These samples represented employees of three types of organization in Russia, namely: (1) Sample 1 dealt with eleven commercial large- and medium-size companies in the field of engineering, dairy products, sale of petroleum industry products, automobiles, and other goods, construction, informational technologies (IT) and real estate (*N* = 198 employees), and one regional institution (state type of ownership) specialized in providing social services to the population (*N* = 69); (2) Sample 2 included staff of one regional division of a state law enforcement agency—Federal Penitentiary Service of Russia (*N* = 278); and (3) Sample 3 was composed of academic professionals from seven state higher education institutions (*N* = 183).

In data processing, incomplete answer sheets from the participants were discarded, thus resulting in sample reduction down to 678 participants, of which: (a) 241 employees in Sample 1—29.0% men and 71.0% women, aged between 24 and 68 (*M* = 38.7; *SD* = 9, 03) with average work experience in the organization/in its structural unit (group) of 6.51/5.57 years, respectively; (b) 265 employees in Sample 2 (76.6% men and 23.4% women) with an age range of 19–55 (*M* = 32.4; *SD* = 6.97) and the average length of service in the organization/in its structural unit (group) 7.3/5.6 years, respectively; and (c) in Sample 3, 176 employees (33.9% men and 66.1% women), aged between 23 and 75 (*M* = 43.8; *SD* = 11.8) with an average work experience in the institution/in its structural unit (group) of 13.9/11.2 years, respectively. Respective sample sizes are considered to be sufficient as estimated by G*-power calculator for correlational analyses with the estimated effect size of 0.3 (average observed in previous studies) and power set at 0.85 (ɑ = 0.05).

Within the socio-economic sphere (companies and the social welfare institution), the study predominantly dealt with office employees. Most of the studied small work groups, of which the participants were members, are characterized by modest work co-dependence with a focus on individual results and personal responsibility. Within-group interactions are relatively high in both regularity and intensity. Study participants from the law enforcement agency were mainly the officers of the regime and security services in charge of the defendants and prison inmates (approximately 85% of all participants) and the administrative staff (roughly 15%). This type of organization is typically characterized by predetermined vertical subordination of employees, fixed chain of command, unquestionably following the orders of superiors, etc. In addition, the work of the regime and security officers and prison guards is highly interdependent. In groups with highly regular interactions, employees’ actions are driven not only by personal, but also by collective responsibility. In higher education, the participants were exclusively academic professionals (professors, associate professors, senior teachers and lecturers). Their professional activities are obviously quite individual and creative in nature; accordingly, participants in this sample are personally responsible for their job performance. Within the university departments (as approximated small groups), the regularity of interactions among academic staff is relatively low. Democratic atmosphere (equal-footing interactions) is the most prevalent in institutions of higher education.

Thus, it is quite obvious that the samples in our study differed substantially in their respective institutional characteristics, content of professional activity, degree of interdependence of tasks and regularity of interactions.

### 4.2. Measures

The Personal Identification Questionnaire (PIQ), the Interpersonal Identification Questionnaire (IPIQ), the Group and Micro-group Identification Questionnaire (GMGIQ), and the Organizational and Sub-Organizational Identification Questionnaire (OSOIQ) were used [69]. All questionnaires are composed of six items, and include two subscales (cognitive and affective), with three items each. Two of these questionnaires are divided into two parts. GMGIQ includes sections entitled “Group/team as a whole” (for measuring group identification) and “Community of colleagues with whom I maintain friendly relations” (for evaluating micro-group identification). Similarly, OSOIQ features two parts: “Organization [Company, Enterprise]” (for assessing organizational identification) and “Division [Department, Section]” (for studying sub-organizational identification). The only exception to this is the assessment of the law enforcement officials, in which the OSOIQ only used one section for measuring organizational identification and did not have a section for sub-organizational identification. Both parts of each of these questionnaires have a uniform structure (that is the number and the content of items), which made it possible to achieve maximum unification of the tools for assessing group and micro-group identifications, as well as organizational and sub-organizational identifications. Instruction for participants and a sample form of the GMGIQ are provided in the Appendix A. OSOIQ has a similar instruction and form, but different names for its parts: “Organization [Company, Enterprise]” and “Division [Department, Section]”.

Here are some examples of items that compose the scale of cognitive identification: “I feel I am a part of the whole (division/organization)” (OSOIQ), “I perceive common successes or failures as my own” (GMGIQ), “Often I think the same way as some other colleagues” (IPIQ), “I feel that I perceive a lot of things differently from how most my colleagues perceive them”(PIQ); and the scale of affective identification: “As a rule, I am pleased to realize that I work in this collective” (OSOIQ), “As a rule, I enjoy being ain a collective” (GMGIQ), “I feel frustrated, when I cannot reach mutual understanding with those who interest me” (IPIQ), “Often I am more concerned about my own successes than about successes of my colleagues or the of team”(PIQ).

The following values of Cronbach’s α characterize these subscales across the set of the instruments used. For the scale of “cognitive identification”, they were: 0.83 (PIQ); 0.78 (IPIQ); 0.75 and 0.74 (GMGIQ, for its two sections, respectively); 0.75 and 0.79 (OSOIQ, also for the two sections). For the scale of “affective identification”, these values were: 0.71 (PIQ); 0.79 (IPIQ); 0.81 and 0.79 (for the two sections of the GMGIQ); 0.78 and 0.77 (for the two sections of the OSOIQ).

### 4.3. Procedure

All three samples followed the same standard protocol. Before participating in the study sample, all respondents were informed about its objectives. The study was carried out at the workplaces in agreement with the administration of the respective organizations and with the consent of the participants expressed orally. Respondents were given the opportunity to fill out questionnaires on paper using the above-described assessment instruments and the corresponding answer forms, distributed by the researcher, who then collected the completed questionnaires. Employees were informed that their data would be kept confidential to ensure anonymity and only used for research purposes. Of all of the employees who were asked to take part in the study, only eleven declined.

### 4.4. Analyses

Data processing was carried out separately for each category of employee in the socio-economic, law enforcement and educational fields to account for the potential influence of specific characteristics, working conditions and content of professional activities in these areas on how employees realize, perceive and manifest their IDs.

To assess the degree of the difference in ID foci for each of its two dimensions (H1) and, conversely, between the two dimensions at each ID foci (H2), the respective means, and subsequently non-parametric Mann–Whitney criteria, were calculated. To identify significant relationships among ID foci separately for the cognitive and affective dimensions (H3 and H4), Pearson product-moment correlations were used.

In order to verify the factor structure of the set of employees’ identifications in the organization (H5), a confirmatory factor analysis was performed using *AMOS SPSS Version 21*. All calculations were performed using the Asymptotic Distribution-Free (ADF) algorithm. It was assumed that the formation of the structural units of the employee’s identifications in the organization occurs primarily in terms of foci, and not dimensions of identification. Six potential models were tested and compared (as the three samples derived from different professional fields could markedly differ from one another). These are outlined below.

A. Two-factor Model 1: [personal cognitive and affective ID]/[interpersonal cognitive and affective ID—micro-group cognitive and affective ID—group cognitive and affective ID—sub-organizational cognitive and affective ID—organizational cognitive and affective ID].
B. Three-factor Model 2: [personal cognitive and affective ID]/[interpersonal cognitive and affective ID]/[micro-group cognitive and affective ID—group cognitive and affective ID—sub-organizational cognitive and affective ID—organizational cognitive and affective ID].
C. Four-factor Model 3: [personal cognitive and affective ID]/[interpersonal cognitive and affective ID]/[micro-group cognitive and affective ID]/[group cognitive and affective ID—sub-organizational cognitive and affective ID—organizational cognitive and affective ID].
D. Four-factor Model 4: [personal cognitive and affective ID]/[interpersonal cognitive and affective ID]/[micro-group cognitive and affective ID—group cognitive and affective ID]/[sub-organizational cognitive and affective ID—organizational cognitive and affective ID].
E. Five-factor Model 5: [personal cognitive and affective ID]/[interpersonal cognitive and affective ID]/[micro-group cognitive and affective ID]/[group cognitive and affective ID]/[sub-organizational cognitive and affective ID—organizational cognitive and affective ID].
F. Six-factor Model 6: [personal cognitive and affective ID]/[interpersonal cognitive and affective ID]/[micro-group cognitive and affective ID]/[group cognitive and affective ID]/[sub-organizational cognitive and affective ID]/[organizational cognitive and affective ID].

To assess the adequacy of these models, goodness-of-fit statistics were calculated for each of them.

## 5. Results

### 5.1. Relative Strength of ID Foci and Dimensions

Table 1 below summarizes descriptive statistics and cross-correlations of all variables across all three independent samples. Our research Hypothesis 1 suggested that the micro-group ID would be the strongest, and the personal ID the weakest among other employees’ identifications. A pairwise comparison (Mann–Whitney test) of all identities for each dimension separately (Table 2) showed statistically significant differences between some IDs for both dimensions in all three samples. The average values (Table 1) indicate the direction of the differences, i.e., the larger the average, the stronger the corresponding variable (focus and dimension) of ID. The rank comparisons showed the same differences. The results did not fully confirm H1 for all samples. On the one hand, personal ID in both dimensions, as it was hypothesized, appears to be significantly weaker than other ID foci among employees in all three professional fields. On the other hand, micro-group ID is significantly stronger than other IDs only among law enforcement officials. Among the employees of educational institutions, both micro-group and group IDs are expressed approximately to the same extent, and both are stronger than other IDs. Employees in the socio-economic sphere showed the strongest: (a) micro-group and sub-organizational IDs in the cognitive dimension; (b) all—micro-group, group, sub-organizational and organizational— IDs in the affective dimension.

Additionally, a comparison among the employees of the socio-economic, law enforcement and educational fields (Table 1) showed the following statistically significant differences. The socio-economic and law enforcement spheres differed on the measures of: Personal-Cognitive identity (*Z* = −6.04, *p* < 0.001); Interpersonal-Cognitive identification (*Z* = −6.36, *p* < 0.001); Micro-group-Cognitive identification (*Z* = −5.70, *p* < 0.001), Micro-Group-Affective identification (*Z* = −3.86, *p* < 0.001); and Group-Cognitive identification (*Z* = −3.12, *p* < 0.01). There were statistically significant differences between the socio-economic and educational spheres on the measures of: Personal-Affective identity (*Z* = −4.34, *p* < 0.001); Interpersonal-Cognitive identification (*Z* = −3.59, *p* < 0.001); Sub-organizational-Cognitive identification (*Z* =−3.23, *p* < 0.01); and Organizational-Cognitive identification (*Z* = −3.97, *p* < 0.001). Finally, comparison between the law enforcement and educational spheres revealed statistically significant differences on the measures of: Personal-Cognitive identity (*Z* = −5.99, *p* < 0.001); Personal-Affective identity (Z = −3.83, *p* < 0.001); Interpersonal-Cognitive identification (*Z* = −2.82, *p* < 0.01); Micro-group-Cognitive identification (*Z* = −6.41, *p* < 0.001); Micro-group-Affective identification (*Z* = −4.11, *p* < 0.001); and Organizational-Cognitive identification (*Z* = −5.64, *p* < 0.001).

A comparison of the means (and, accordingly, the mean ranks) showed that the higher education workers, in contrast with the law enforcement officers, had significantly stronger personal cognitive and affective identities, and, in contrast with the workers in the socio-economic sphere, had a stronger personal identity only in its affective component. The workers in the socio-economic sphere had significantly stronger personal cognitive identities than the law enforcement officers. In turn, the latter sample had stronger micro-group cognitive and affective identifications, and also group cognitive identification, compared to both other samples. Higher education workers had significantly weaker sub-organizational and organizational cognitive identifications than employees in other professional areas.

We also hypothesized that the affective dimension, as opposed to the cognitive dimension, would be stronger in all ID foci (H2). In fact, as Table 1 and Table 3 indicate, this difference was found in the foci of: personal ID (in the law enforcement institution); micro-group ID (in the socio-economic and educational spheres); group ID (in all three samples); sub-organizational ID (among higher education employees); and organizational ID (in all three categories of employees). In contrast, the cognitive dimension of interpersonal ID was stronger than the affective dimension in all samples. Thus, H2 was confirmed for all identification foci across the samples, with the exception of interpersonal ID.

### 5.2. The Relationships among Foci of ID and the Structural Organization of IDs

We hypothesized that significant positive relationships both for cognitive and affective dimensions exist; first, between social IDs—organizational, sub-organizational, group, and micro-group (H3a)—and second, between all social IDs and interpersonal ID (H3b). Significant positive relationships among micro-group, group, sub-organizational, and organizational IDs for both dimensions were found across all three samples (Table 1). The strongest relationships were found for the employees of the socio-economic sphere. Thus, H3a is confirmed. Also shown were the significant positive relationships of interpersonal ID (a) with all social IDs (on their cognitive and affective dimensions) in the socio-economic and law enforcement samples (Table 1). In the higher education sample, interpersonal ID was significantly positively correlated with group ID (on both dimensions), micro-group ID (affective dimension), and sub-organizational ID (cognitive dimension). In this sample, interpersonal ID was not significantly related to organizational ID. Thus, H3b is fully confirmed in two samples and partially (for three out of four social ID foci) in one sample.

There was also a hypothesis that the relationship of personal ID with all other IDs depends on the professional specialization of employees and on the identification dimensions (H4). It was found that personal ID is significantly negatively connected to all other foci of ID, but only among employees of the law enforcement institution (with the sole exception of organizational-cognitive ID, Table 1). The employees in the socio-economic sphere demonstrated significant negative relationships between personal ID, and only interpersonal ID in the affective dimension, whereas no significant relationships were discovered in the other cases. Among higher education employees, personal ID exhibited a significant negative relationship only with interpersonal ID in the cognitive dimension, and with micro-group, group, sub-organizational, and organizational IDs in the affective dimension. Therefore, Hypothesis 4 is confirmed.

We suggested that the ID foci (with the two dimensions in each) can form a certain structure in which personal ID will always stand out as a relatively independent unit, whereas the remaining identification foci (also, in their cognitive and affective dimensions simultaneously) will form different configurations in different areas of professional activity (H5). A confirmatory factor analysis yielded the following outcomes (Table 4). The analysis showed that for law enforcement (sample 2), model 6 is the most appropriate among all tested models. The following goodness-of-fit indicators were obtained: χ2 = 56.3; df = 25; *p* < 0.001; CFI = 0.92; RMSEA = 0.06; 90% confidence interval for RMSEA (0.05–0.09). This model showed that all identifications are independent units (with two dimensions in each). It should be noted that sub-organizational ID was not studied in this sample, and therefore it cannot be unambiguously stated whether this identification is an independent unit or is integrated with organizational ID. The goodness of fit indices across other considered models in all three samples did not achieve critical values. Thus, H5 is confirmed only for the law enforcement sample.

In addition, we tested similar models, for each individual identification dimension (cognitive and affective), using confirmatory factor analysis. These twelve models were not statistically significant either.

## 6. Discussion

The much weaker personal ID (in comparison with other ID foci), observed in all three samples of our research project, could be the result of the very fact of the individual’s presence in the collective (whether it is an informal subgroup, small group, unit/division or an entire organization), which implies the primacy of the collective over the individual, i.e., a substantial influence of the collective on its individual members. This assumption is supported by the observed differences between the qualitative descriptions of the participants in their group identity and their personal identity [70]. Values, emotions and personal relationships were more often listed in representations of group identity than in representations of personal identity. Indeed, the activity of an employee in the organization (group, etc.) should, first of all, correspond to the key attributes of this collective actor, rather than the desire to show his unique personal qualities. If the employee constantly strives to show his individuality, which is not consistent with the expectations (in relation to him) of the collective actor, it may lead to a conflict between the employee and the collective actor, to deterioration in this person’s psychological status, to a lesser acceptance by others, etc. However, the actual salience of personal ID may vary and, as shown by the example of our samples, depends on the specific organizational environments.

Additionally, it would be plausible to assume that many employees in the workplace often unconsciously “suppress” their personal ID. Showing initiative, publicly expressing opinions on work issues that differ from the opinions of their colleagues, defending one’s own opinion in a dispute with a manager, etc., are all manifestations of employees’ personal identity, and not just of certain types of personality traits. These activities reveal the unique characteristics of individuals. Many employees may be afraid to display such behaviors to protect themselves from potential problems of added responsibilities, mistreatment by colleagues, conflict with and retaliation from the leader, to name just a few. Lay “wisdom” reflects this cautious attitude in a number of fabled sayings, for example, “the boss is always right”, “showing initiative may be punishable”, “it is safer to follow the herd than to lead it”. As a result, many employees may constrain their personal ID, specifically in professional contexts—it would be misleading to think that the collective pressure and/or individual’s caution suppress all aspects of personal ID. Employees may still have quite a noticeable personal ID in those moments that do not relate directly to work requirements and regulations. For example, some women who wear corporate or military uniforms tend to highlight their uniqueness through various small, but noticeable, additional elements in clothing, through hairstyle, use of para- and extra-linguistic means, etc. In other words, for many people, depending on external circumstances, some facets of personal ID (related to professional, personal, physical and other unique qualities) can either be actualized or remain merely potential. That would suggest looking at personal ID (perhaps, to an extent, also at social IDs) as a psychological phenomenon that has the property of partiality.

The apparent predominance of micro-group ID over other IDs (most clearly in two samples) could be explained as follows. In small groups, stable informal subgroups with distinct boundaries are formed. Within those, very close relationships are easily established, high interaction intensity is achieved, and solidarity and trust exist to a much larger extent than in a group, division, or an entire organization. For members of a certain informal subgroup and even for some employees not included in any subgroup, a subgroup may still be more referent (significant) than other structural units. Hence, the behavior of members of informal subgroups, at least within an in-group, depends primarily on their identification with this subgroup, and not on their identification with either the group or the entire organization. Of course, micro-group ID determines the behavior of the members of the subgroup not in all situations, but in those where the goals and interests of the subgroup as a collective actor are affected. This, in fact, has not been taken into account in most studies of the behavioral consequences of social (organizational, group) IDs. In addition, some questions arise that require a more detailed study, for example: Under what conditions, and how, does the salience of micro-group and group (sub-organizational, organizational) IDs simultaneously change (strengthen/weaken)? How and under what conditions does a certain degree of the micro-group ID on the one hand, and the group ID on the other, affect the contribution of individual members to group work?

Thus, what all samples have in common is the lower strength of personal ID and the greater salience of micro-group ID. These results (depending on the sample and some specific characteristics of their professional activities) are consistent with the studies in which micro-group ID was stronger than group ID [18], as was group ID compared to personal ID [70].

Some differences between dimensions were, indeed, observed in the study. In their relationships with the colleagues who employees identify themselves with (i.e., interpersonal ID), the perceptual (cognitive) component is somewhat more relevant than the emotional (affective) one. In other words, perception of others through the prism of significant characteristics that they possess, and shared backgrounds, creates a sense of community and involvement with others. In the employees’ attitudes towards themselves, a subgroup, group, division and organization, the affective dimension, on the contrary, is more significant, as it reflects experiencing feelings of connection to oneself/structural unit and of events happening to oneself/structural unit. This described difference could be explained by the fact that a collective, in contrast to an individual, does not possess personal characteristics to which a person may relate and/or strive to achieve for oneself. By and large, people are more inclined, on the one hand, to experience connections with the collective (affective dimension), and on the other hand, to perceive connections with other individuals (cognitive dimension). It is probably more difficult for people to comprehend a collective actor than another individual, but it is easier to experience a connection with it. This makes one think about the validity of the opinion about the purely cognitive nature of social (organizational, group) IDs, which is often found in the literature [53,71,72]. How often do we actually impartially analyze the teams (work group, organization, company of friends, etc.) in which we are included? Can we confidently answer questions such as: What are the norms and values of this team? What is this team capable of in difficult situations?

However, this explanation does not fully fit personal ID, which is characterized by the predominance of the affective dimension over the cognitive one. The latter could probably be explained by the fact that most people are more inclined towards experiencing authenticity than to critical self-reflection. Unfortunately, we could not locate research literature that would compare relative strength of ID dimension for a specific ID focus, so it is difficult to put our results in a related context. It is why the question of predominance of the affective component over the cognitive component of personal ID deserves special investigation and careful conceptual reflection at the junction with psychology of personality.

All social IDs were found to be significantly positively correlated with each other. Major arguments that could explain significant positive connections among all foci of social ID include: (1) the fact that all employees are, at the same time, involved with their respective small groups, secondary-level structural divisions and organizations that compose a hierarchical structure (group-division-organization); and (2) that many employees are also included in various informal subgroups or that certain subgroups serve as referent groups for those who are not included in any subgroup. This reflects the idea of the plurality of social IDs, i.e., nested and cross-cutting social IDs [73,74].

In turn, interpersonal ID was significantly positively correlated with all social IDs (in both dimensions) in socio-economic and law enforcement samples, as well as with group ID (in both dimensions), with micro-group ID and sub-organizational ID (in one dimension). As we have previously suggested, the identification of employees with colleagues is probably projected onto their identification with the corresponding structural units. For instance, if employee A is identified with employee B, who identifies himself with a certain structural unit, then it is possible that employee A will be identified with this unit too. Conversely, the identification of workers with structural units may be the basis for their interpersonal ID. These data are consistent with the studies that found associations among some social ID foci [15,34,54] and between some social IDs and interpersonal IDs [37,63].

The relationship of employees’ personal ID with other IDs, in many ways depends on the specifics of a particular professional sphere and the ID dimension. For instance, an important role in the correlation between personal ID and social ID can be played by such job characteristics as: rigid vertical subordination or, on the contrary, flexible functional connections in the organization; on complex (creative) or executive tasks; high or low degree of tasks’ interdependence; high or low regularity and intensity of interactions; high or low expectations for employees to show individual initiative; on the prevalence of personal or collective responsibility; and on the priority of either individual or group results. Our research shows that, depending on some of the above-listed contextual and interactive characteristics of the samples in Samples 1–3, there can be either a negative relationship (relatively speaking, mutual exclusion) or a lack of connection (simultaneous manifestation of both) between personal ID and other IDs. Thus, the question of under what conditions personal ID and social IDs complement [67], mutually exclude [26], or just compete with each other [32], remains open for further research.

Confirmatory factor analysis showed various degrees of difference in the structure of employees’ IDs dependent on a specific professional field. However, only the mode based on data from the law enforcement sample showed acceptable ‘goodness of fit’ parameters. In this model, all identities were relatively independent structural units. Moreover, the ID foci, but not the dimensions of ID, appeared to be the leading factors in the formation of the structure of IDs. In other words, each observed factor includes both ID dimensions. Unfortunately, we cannot compare these results with any relevant data from other studies, as those were not found.

### Theoretical and Practical Implications

The results of this research expand our understanding of the IDs of employees in an organization: (1) about those ID foci that previously have not been sufficiently studied (for example, micro-group ID); (2) about the relative strength of a wide spectrum of employees’ IDs with collective and individual actors; (3) about the relative strength of two dimensions of each ID; and (4) about the relationships between different foci of ID, as well as about the structure of IDs in general. Together, these findings produce a holistic picture of employees’ identifications in an organization and deepen our conceptual understanding of the components of self-concept. Our comprehensive study of three different samples made it possible to identify some of the general and specific trends in various IDs’ salience and the associations among them. It can be assumed that the common features we found here may be present in other samples that differ in terms of their professional activities. If this is confirmed in future research, the relevant facts and implications may acquire universal scientific significance.

This investigation has, however, some practical implications. Knowledge of convexity and the relationships of a wide range of ID foci and their dimensions, gained through this research, can contribute to the better understanding of organizational attitudes and behaviors. For example, micro-group identification, compared to other social IDs, will be a stronger predictor of organizational citizenship behaviors. The higher strength of the affective dimension of some social IDs makes it more likely to lead to stronger association with such behaviors compared to the cognitive dimension of the respective IDs. The relative salience not only of organizational and group identifications, but also of group and micro-group identifications, group and personal identifications, etc., could create a noticeable effect on manifestations of citizenship behavior. Knowing this will help Human Resources management to suggest adequate measures for increasing a certain ID by either or both of its dimensions. In addition, managers must consider content and specific working conditions in order to strike an optimal balance between personal ID and a particular focus of social ID. Managers can involve employees in the decision-making processes at the level of a group, unit or an entire organization and develop measures to increase primarily the corresponding focus of ID.

## 7. Conclusions

This study is one of the rare attempts to understand the connections and relationships among different ID foci (i.e., personal, interpersonal, micro-group, group, sub-organizational and organizational) across two dimensions (cognitive and affective) of participating employees from three different professional fields—socio-economic, law enforcement, and higher education. It was found that the strength of some IDs and the relationships among them depend on specific work characteristics. However, there are also stable trends irrespective of the professional field. Those are: (1) the fact that personal ID is weaker, whereas micro-group ID is stronger, than the rest of the IDs (in both dimensions); (2) there are positive connections among the foci of social ID (in both dimensions); and (3) the affective dimension prevails over the cognitive one in micro-group, group and organizational IDs, and, conversely, the cognitive dimension is more prominent in interpersonal ID.

This study, for the first time, examines simultaneously all possible foci of employees’ ID in order to provide a theoretical explanation of the connections and relationships among them. Since our results are limited to certain professional samples in a Russian context only, they cannot be generalized conclusively. Further research is undoubtedly needed to support our findings and their implications for research and practice (e.g., on samples from other professional fields—with their respective job characteristics—and in other countries).

### Limitations and Future Research

This research has some limitations. The sample of employees in the socio-economic sphere was not homogeneous, as some organizations differed markedly in the profile of their professional activity. Additionally, the study did not take into account the character of interactions (competition, cooperation or lack of active interaction) between groups and units in the organization, which could affect the strength of the corresponding IDs and the relationships among them.

In light of the findings of the current study, especially paying attention to different foci of the employees’ ID, several prospects for future research are open. First, it is necessary to more thoroughly study the role that specific characteristics of jobs and working conditions play in the degrees of manifestation and proportionality (quantitative ratio) of various IDs for each of the two ID dimensions, and in the relationships among IDs and their respective structures.

Second, it is important: (a) to study the property of partiality of personal ID depending on the unique characteristics of a person (professional, personal, physical, etc.), as well as this property of various social IDs; (b) to analyze the conditions and mechanism of the interconnected dynamics (strengthening and weakening) of social IDs—micro-group, group and organizational, as well as personal ID; and (c) to comprehend the reasons for the predominance of the affective component over the cognitive component of social IDs and personal ID.

Third, various IDs can have different effects on certain attitudes, behaviours, and work performance. In addition, they may have differing effects on different organizational strata, such as groups, divisions and the whole organization. For example, in a meta-analysis of attachment correlation studies (i.e., identification and commitment), group attachment was found to correlate with extra-role behaviour at the workgroup level more strongly than organizational attachment [21]. On the other hand, group attachment, as compared to organizational attachment, showed weaker correlations with extra-role behaviour in the organizational context. Therefore, the effects of each ID focus need to be considered not only in general, but also in specific contexts.

Researchers often study the isolated effects of ID (usually, organizational and group). However, different ID foci can create substantial interaction effects. Therefore, it is necessary to study the consequences of each focus of ID not in isolation, but the effects of certain ID foci combined in various proportions (e.g., personal/group, micro-group/group).

## Figures and Tables

**Table 1:** Descriptive Statistics and Inter-correlations Among Variables.

Variable	Samples:			Cognitive Dimension					Affective Dimension						
	1–3	*M*	*SD*	2	3	4	5	6	*M*	*SD*	2	3	4	5	6
1. PI	S-E	9.02	3.22	0.07	0.03	0.07	0.09	0.00	8.97	3.11	−0.27 **	−0.05	−0.08	−0.08	−0.02
	L-E	7.28	3.59	−0.30 ***	−0.14 *	−0.15 *	-	−0.07	8.98	3.85	−0.15 *	−0.14 *	−0.21 ***	-	−0.16 **
	H-E	9.50	3.86	−0.22 **	−0.13	0.05	−0.14	−0.07	10.26	3.35	−0.11	−0.16 *	−0.20 **	−0.29 ***	−0.26 ***
2. II	S-E	11.47	3.00		0.41 ***	0.42 ***	0.52 ***	0.33 ***	10.70	3.47		0.32 ***	0.28 ***	0.34 ***	0.20 **
	L-E	13.31	3.39		0.35 ***	0.21 **	-	0.34 ***	10.99	4.09		0.18 **	0.13 *	-	0.24 ***
	H-E	12.58	2.79		0.07	0.16 *	0.22 **	0.11	11.09	3.05		0.15 *	0.17 *	0.11	0.12
3. MgI	S-E	13.22	3.40			0.76 ***	0.71 ***	0.53 ***	13.85	3.60			0.81 ***	0.72 ***	0.64 ***
	L-E	14.89	2.50			0.39 ***	-	0.22 **	15.20	2.55			0.39 ***	-	0.16 **
	H-E	13.32	2.64			0.48 ***	0.30 ***	0.30 ***	14.11	2.88			0.61 ***	0.44 ***	0.40 ***
4. GI	S-E	12.67	3.24				0.74 ***	0.52 ***	13.73	3.36				0.70 ***	0.68 ***
	L-E	13.55	3.00				-	0.49 ***	14.22	3.21				-	0.36 ***
	H-E	12.97	3.22				0.27 ***	0.28 ***	14.25	3.14				0.45 ***	0.49 ***
5. SoI	S-E	13.23	3.36					0.72 ***	13.77	3.31					0.80 ***
	L-E	-	-					-	-	-					-
	H-E	12.18	3.37					0.77 ***	13.32	3.10					0.79 ***
6. OI	S-E	12.50	3.11						13.87	3.06					
	L-E	13.06	3.20						13.65	3.35					
	H-E	11.27	3.36						13.18	3.46					

Identification Foci: PI—personal ID, II—interpersonal ID, MgI—micro-group ID, GI—group ID, SoI—sub-organizational ID, and OI—organizational ID. Professional Fields: Study 1: S-E—Socio-Economic; Study 2: L-E—Law Enforcement; Study 3: H-E—Higher Education. * *p* < 0.05; ** *p* < 0.01; *** *p* < 0.001.

**Table 2:** Comparison of ID Foci for Each Dimension Individually.

ID Dimensions	Sample:	ID Foci														
	1–3	PI/II	PI/MoI	PI/GI	PI/SoI	PI/OI	II/MoI	II/GI	II/SoI	II/OI	MoI/GI	MgI/SoI	MgI/OI	GI/SoI	GI/OI	SoI/OI
Cognitive	S-E	−8.42 ***	−11.90 ***	−10.99 ***	−11.66 ***	−10.88 ***	−6.27 ***	−4.42 ***	−6.07 ***	−3.90 ***	−2.10 *	−0.01	−2.83 **	−2.00 *	−0.71	−2.72 **
	L-E	−15.07 ***	−17.63 ***	−15.81 ***	-	−14.96 ***	−5.36 ***	−0.63	-	−1.03	−5.17 ***	-	−6.84 ***	-	−1.73	-
	H-E	−7.50 ***	−9.23 ***	−8.10 ***	−6.37 ***	−4.25 **	−2.83 **	−1.61	−0.92	−3.84 ***	−0.79	−3.33 ***	−6.20 ***	−2.24 *	−4.83 ***	−2.64 **
Affective	S-E	−5.72 ***	−12.86 ***	−13.05 ***	−12.88 ***	−13.83 ***	−8.21 ***	−8.85 ***	−8.97 ***	−9.57 ***	−0.76	−0.70	−0.72	−0.24	−0.24	−0.03
	L-E	−5.50 ***	−15.91 ***	−13.73 ***	-	−12.55 ***	−11.82 ***	−9.09 ***	-	−7.42 ***	−3.72 ***	-	−5.48 ***	-	−2.01 *	-
	H-E	−2.21 *	−9.98 ***	−10.04 ***	−7.90 ***	−7.24 ***	−8.60 ***	−8.78 ***	−6.29 ***	−5.68 ***	−0.81	−2.43 *	−2.45 *	−3.06 **	−2.97 **	−0.16

* *p* < 0.05; ** *p* < 0.01; *** *p* < 0.001. Weighted average effect sizes (expressed as Hedges’ g), estimated from statistical significance values of non-parametric assessment of difference between specific foci of employees’ identifications, were as follows. S-E sample: 0.034 and 0.29—for cognitive and affective dimensions, respectively. L-E sample: 0.21 and 0.26. H-E sample: 0.39 and 0.35.

**Table 3:** Comparison of Cognitive and Affective Dimensions for Each ID Focus.

ID Foci	ID Dimensions Comparison (Cognitive/Affective)	
	Sample	*Z*
Personal	SE	−0.16
	LE	−5.26 ***
	HE	−1.97
Interpersonal	SE	−2.47 *
	LE	−6.52 ***
	HE	−4.47 ***
Micro-group	SE	−2.54 *
	LE	−1.77
	HE	−2.75 **
Group	SE	−3.50 ***
	LE	−2.94 **
	HE	−3.87 ***
Sub-organizational	SE	−1.77
	LE	-
	HE	−3.06 **
Organizational	SE	−4.74 ***
	LE	−2.39 *
	HE	−5.03 ***

* *p* < 0.05; ** *p* < 0.01; *** *p* < 0.001.

**Table 4:** Goodness of Fit Statistics for Three Samples’ Models.

Models	Sample: 1–3	*df*	*χ^2^*	CFI	RMSEA	RMSEA 90% *Confidence Interval*	*p*
1	S-E	62	505.2	0.20	0.18	0.17–0.20	0.00
	L-E	34	172.3	0.65	0.13	0.11–0.14	0.00
	H-E	53	204.7	0.70	0.13	0.01–0.15	0.00
2	S-E	60	305.1	0.56	0.14	0.12–0.15	0.00
	L-E	32	139.4	0.73	0.11	0.09–0.13	0.00
	H-E	51	200.9	0.71	0.13	0.11–0.15	0.00
3	S-E	56	251.8	0.65	0.13	0.11–0.14	0.00
	L-E	29	103.8	0.81	0.10	0.07–0.12	0.00
	H-E	48	171.9	0.76	0.12	0.10–0.14	0.00
4	S-E	56	267.0	0.62	0.13	0.12–0.15	0.00
	L-E	29	84.4	0.86	0.09	0.06–0.11	0.00
	H-E	48	157.3	0.79	0.11	0.10–0.13	0.00
5	S-E	51	202.5	0.73	0.12	0.10–0.13	0.00
	L-E	-	-	-	-	-	-
	H-E	44	131.4	0.83	0.11	0.08–0.13	0.00
6	S-E	45	175.6	0.77	0.12	0.10–0.13	0.00
	L-E	25	56.3	0.92	0.06	0.05–0.09	0.00
	H-E	45	157.5	0.78	0.12	0.10–0.14	0.00

## Data Availability

The datasets generated during and/or analysed during the current study are available in the Figshare repository (doi:10.6084/M9.figshare.18598721), accessed on 18 January 2022.

## Appendix A. Instruction for Participants and Sample Form of the Group and Micro-Group Identification Questionnaire (GMGIQ)

INSTRUCTION: Read the six items below and evaluate to what extent they reflect the reality of: (1) your group/team, for example department, brigade, shift (on the right side of the items); (2) the community of those with whom you maintain friendly relationships within your group/team (on the left side of the items). For the assessment use the six-point scale, where “1” means “strongly disagree”, “6” means “strongly agree”, and the remaining points in between express different degrees (less strong) of your agreement/disagreement with the statement. So, for each item, please, select (circle or checkmark) one number that most accurately reflects your opinion.

**Table A1:** Micro-group Identification Questionnaire (GMGIQ).

Community of Colleagues with Whom I Maintain Friendly Relations						Examples of the Questionnaire Items	Group/Team as a Whole					
1	2	3	4	5	6	I feel I am a part of the whole	1	2	3	4	5	6
1	2	3	4	5	6	I perceive common successes or failures as my own	1	2	3	4	5	6
